# Transcending Age Barriers: Successful Management of Pediatric Dilated Cardiomyopathy with Rare PLEKHM2 Mutation in an Adult Hospital

**DOI:** 10.1016/j.jaccas.2024.103023

**Published:** 2025-03-05

**Authors:** Solomon Bendayan, Elie Ganni, Maria Victoria Ordonez, Ethan Bendayan, Yossi Cohen, Gordon Samoukovic, Nadia Giannetti

**Affiliations:** a Department of Internal Medicine, McGill University, Montreal, Quebec, Canada; b Department of Cardiology, McGill University, Montreal, Quebec, Canada; c Faculty of Medicine and Health Sciences, McGill University, Montreal, Quebec, Canada; d Department of Critical Care, McGill University, Montreal, Quebec, Canada

**Keywords:** advanced heart failure therapies, genetic testing, multidisciplinary management, pediatric dilated cardiomyopathy, PLEKHM2 mutation

## Abstract

This case report describes a complex presentation of dilated cardiomyopathy (DCM) in a 14-year-old boy of Indian origin, initially presenting with nonspecific abdominal pain, who was eventually found to have severe biventricular dilatation and a rare genetic mutation in PLEKHM2, associated with increased trabeculations and DCM. His condition rapidly progressed to critical cardiogenic shock, necessitating advanced heart failure therapies. This case emphasizes the importance of considering DCM in pediatric patients with atypical presentations and underscores the utility of genetic testing in identifying rare pathologic conditions. It also highlights the challenges and successful management strategies in a pediatric patient treated within an adult health care setting, demonstrating the vital role of tailored multidisciplinary approaches in managing complex cardiomyopathies. The findings contribute to the limited literature on PLEKHM2-associated cardiomyopathy.

## Graphical Abstract

**Figure:**
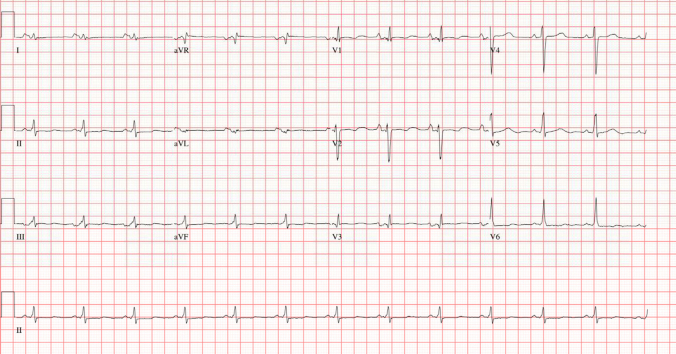

A teenaged boy, 14 years old, originally from India, presented to the pediatric emergency department because of abdominal pain lasting 1 day. The results of physical examination were remarkable for mild epigastric pain on deep palpation without guarding or rebound tenderness. He was discharged home with empiric proton pump inhibitor therapy. He returned to the emergency department 3 weeks later with right upper quadrant and right flank pain. At this point, a review of symptoms was significant for 3 weeks of shortness of breath on exertion (NYHA functional class II). Abdominal examination this time revealed right upper quadrant tenderness, and cardiac examination revealed a grade II/VI holosystolic murmur best heard at the apex.

**Take-Home Messages**

• This case highlights the challenges of treating pediatric patients with adult-like dimensions, demonstrating the importance of tailored multidisciplinary care.
• The patient's progression from heart failure to critical cardiogenic shock and the rare PLEKHM2 mutation underscore the complexity of advanced heart failure therapies, including extracorporeal membrane oxygenation, LVAD, and heart transplantation.

## Past Medical History

His past medical history was noncontributory, and no predisposing risk factors were present. Additionally, there was no family history of cardiac disease or sudden cardiac death.

## Differential Diagnosis

On the basis of the patient's recurring abdominal pain, and initial presentation with epigastric pain, the differential diagnoses included peptic ulcer disease, cholelithiasis or cholecystitis, hepatitis, pancreatitis, and atypical appendicitis, in which case the murmur discovered on physical examination would be considered unrelated to the patient’s presentation of abdominal pain. However, given the cardiac murmur, conditions such as endocarditis, myocarditis, and other cardiomyopathies should also have been considered. We suspected that the patient’s presentation of abdominal pain was secondary to hepatic congestion from heart failure.

## Investigations

**Figure 1:**
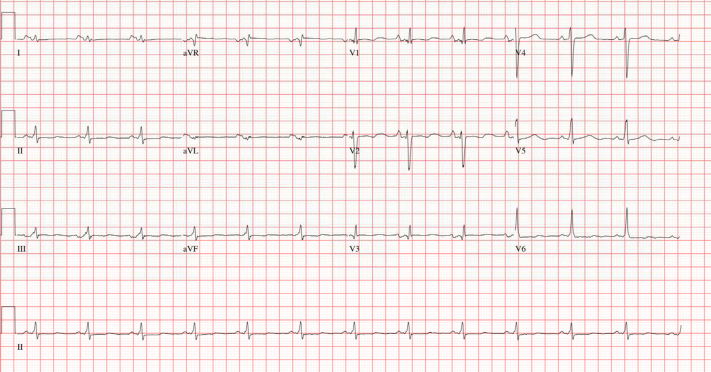
Electrocardiogram Electrocardiogram shows normal sinus rhythm at 74 beats/min, right ventricular hypertrophy, and prolonged QTc of 497 ms.

**Figure 2:**
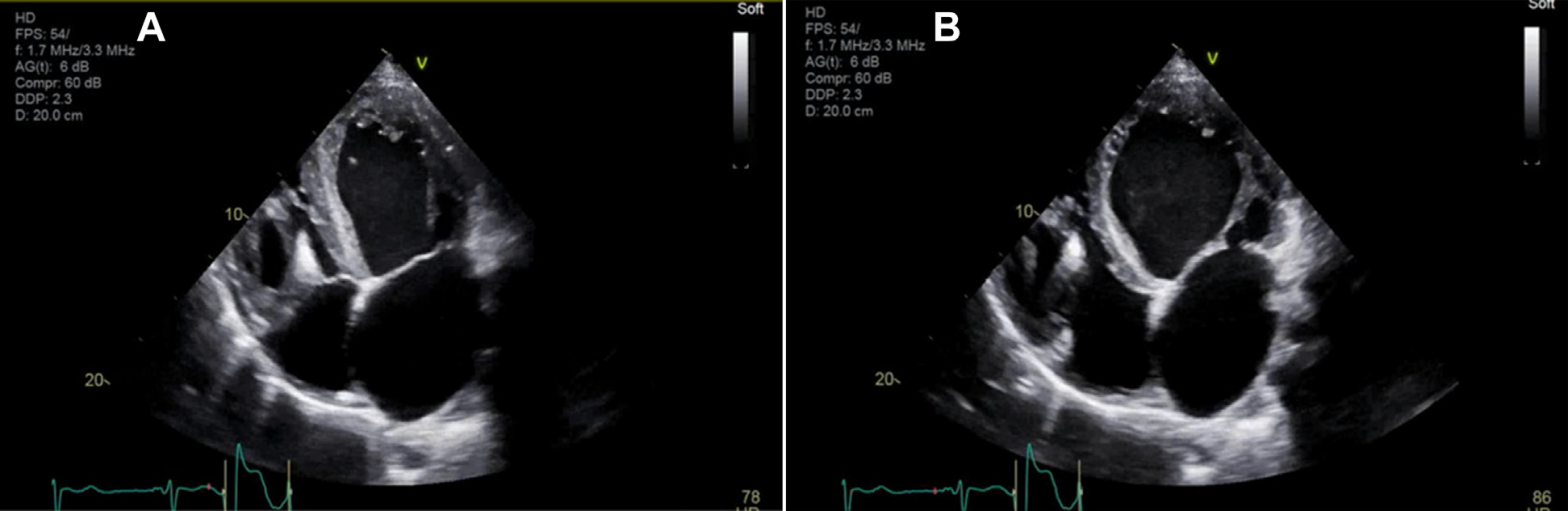
Transthoracic Echocardiogram (A) 4-chamber view in systole reveals a left ventricular ejection fraction biplane of 26% and a hypertrabeculated left ventricle with severe biventricular and left atrial dilatation. (B) 4-chamber view in diastole reveals a left ventricular ejection fraction biplane of 26% and a hypertrabeculated left ventricle with severe biventricular and left atrial dilatation.

A chest radiograph revealed an enlarged cardiac silhouette, and ultrasonography of the abdomen revealed increased periportal echogenicity. The electrocardiogram displayed right ventricular hypertrophy and a prolonged QTc of 497 ms (Figure 1). A transthoracic echocardiogram demonstrated severe functional mitral regurgitation, left ventricular ejection fraction (LVEF) biplane of 26%, and a hypertrabeculated left ventricle with severe biventricular and left atrial dilatation (Figures 2A and 2B). Laboratory testing did not reveal any significant abnormalities. Guideline-directed medical therapy, including a beta-blocker, an angiotensin-converting enzyme inhibitor, and a mineralocorticoid receptor antagonist, was initiated. The patient was subsequently discharged with a plan for close outpatient follow-up at a specialized pediatric heart failure center.

A diagnostic workup was performed in accordance with the 2022 AHA/ACC/HFSA Guideline for the Management of Heart Failure.^1^ The patient underwent systematic evaluation using a multiparametric approach. including clinical evaluation, pedigree analysis, Holter monitoring, laboratory tests, and multimodality imaging with echocardiography and cardiac magnetic resonance. Laboratory testing recommended for all patients with newly diagnosed cardiomyopathy, such as thyroid-stimulating hormone levels, and iron studies did not reveal a cause of his condition. N-terminal pro–B-type natriuretic peptide was elevated at 3,042 pg/mL.

**Figure 3:**
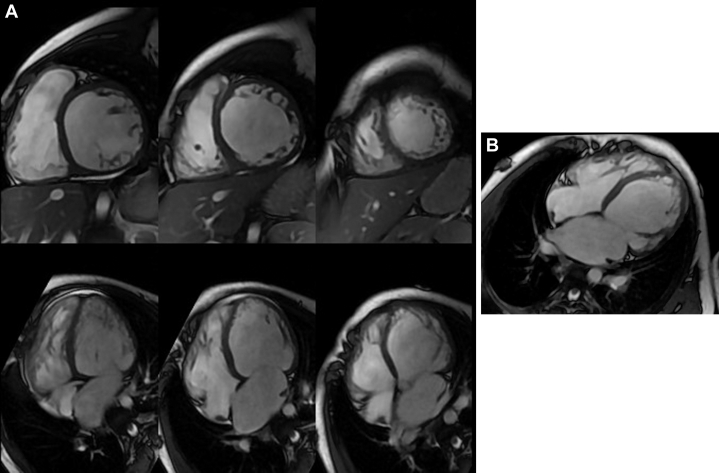
Cardiac Magnetic Resonance Image shows a dilatated and hypertrabeculated left ventricle with left ventricular ejection fraction of 23% and a dilated right ventricle with decreased right ventricular ejection fraction (27%). (B) Image shows a small area of delayed enhancement at the level of the anterior septum.

Cardiac magnetic resonance revealed a dilated and hypertrabeculated left ventricle with LVEF of 23%, dilated right ventricle with decreased right ventricular ejection fraction (27%), and a small area of delayed enhancement at the level of the anterior septum (Figures 3A and 3B).

Genetic testing was performed using next-generation sequencing with a hybridization-based target capture method. The sequencing libraries were prepared and sequenced using Illumina's sequencing-by-synthesis method with paired-end sequencing (150 by 150 bases). The data analysis included alignment to the human reference genome (GRCh37/hg19) and variant calling using genome analysis toolkit algorithms. The identified variants were further validated using Sanger sequencing where necessary. Genetic testing revealed a heterozygous mutation for PLEKHM2 c.2266del, p(Glu756Serfs∗64), which was classified as likely pathogenic, as well as a heterozygous mutation for PLEKHM2 c.1915C>T, p(Arg639Trp), which was classified as a variant of uncertain significance. The patient thus received a diagnosis of genetic hypertrabeculated dilated cardiomyopathy (DCM) associated with PLEKHM2 mutation. To our knowledge, only 2 studies, 1 of which was a case series, have previously described new cases of PLEKHM2-associated DCM.^2^

## Management

Several weeks later, the patient presented to the emergency department with malignant ventricular tachycardia requiring electrical cardioversion. Given that his weight and stature were comparable with that of an adult (65 kg and 181 cm, respectively), there was debate as to whether he should be transferred to a specialized adult or a pediatric heart failure center. Given his tall stature, it is worth noting that the patient did not exhibit typical features consistent with a marfanoid phenotype or a familial predisposition to such conditions. He was ultimately transferred to an adult hospital, given the greater expertise available in advanced mechanical support and heart transplantation, as well as the larger multidisciplinary team available for the care of patients with cardiomyopathies. In keeping with the 2022 AHA/ACC/HFSA Guideline for the Management of Heart Failure,^1^ the patient received high-level multidisciplinary cardiomyopathy care from the following team members: adult and pediatric cardiomyopathy specialists, heart failure team, arrhythmia team, geneticists, surgeons, and patient support resources such as psychologists.

Upon transfer to the adult hospital, the patient was treated with antiarrhythmic therapy, and goal-directed medical therapy was adjusted. He underwent insertion of an implantable cardiac defibrillator and was discharged with outpatient follow-up with the heart failure team, consistent with the 2017 AHA/ACC/HRS guideline for management of patients with ventricular arrhythmias and the prevention of sudden cardiac death, which provides a Class 1B recommendation for insertion of an implantable cardiac defibrillator in patients with hemodynamically unstable ventricular tachycardia not due to reversible causes.^3^

Over the course of the next few months, his clinical symptoms and signs began to deteriorate. Right heart catheterization revealed a cardiac index of 1.6 L/min/m^2^, pulmonary capillary wedge pressure of 29 mm Hg, mean pulmonary artery pressure of 47 mm Hg, central venous pressure of 12 mm Hg, and a pulmonary vascular resistance of 6.1 Wood units. He was admitted to the adult cardiology ward for inotropic therapy, diuresis, and transplantation workup. During that admission, he experienced refractory ventricular tachycardia. Within hours, his hemodynamic status began to deteriorate, with increasing vasopressor requirements, rising lactate, and deteriorating renal function. Impella insertion was considered; however, a peripheral angiogram demonstrated that his femoral vessels were too small for an Impella microaxial left ventricular assist device. The patient experienced worsening cardiogenic shock, ultimately progressing to critical cardiogenic shock (INTERMACS 1), at which point he was given extracorporeal membrane oxygenation and listed for cardiac transplantation.

## Outcome

After 15 days of extracorporeal membrane oxygenation support but no suitable donor heart, the patient was transitioned to a Levitronix CentriMag left ventricular assist device for intermediate duration support. After 2 weeks, a suitable donor was found, and the patient underwent heart transplantation. He was discharged from the hospital in stable condition several weeks later.

## Conclusions

DCM is characterized by the enlargement of 1 or both ventricles, unrelated to abnormal loading conditions or significant coronary artery disease–induced ventricular remodeling.^4^ Acquired factors or genetic abnormalities, particularly autosomal-dominant inheritance, are implicated in its cause.^5^ PLEKHM2 encodes a pleckstrin homology domain–containing protein involved in autophagy and lysosomal function. Homozygous or compound heterozygous mutations in this gene have been associated with DCM and hypertrabeculation. The patient’s frameshift mutation, c.2266del, p(Glu756Serfs∗64), is likely pathogenic, aligning with previous findings where similar truncating mutations disrupt lysosomal function, leading to cardiomyopathy. The c.1915C>T, p(Arg639Trp) variant, although it is classified as a variant of uncertain significance, has potential pathogenic implications based on in silico predictions and its absence in control populations. The interplay between these variants and their contribution to the patient’s phenotype underscores the complexity of genetic cardiomyopathies.

PLEKHM2 mutation is a rare form of DCM inherited in an autosomal recessive fashion.^6^ To our knowledge, only 2 studies, 1 of which was a case series, have previously described new cases associating homozygous or compound heterozygous PLEKHM2 mutations with DCM and hypertrabeculations.^6^^,^^2^ In one study, Muhammad et al^6^ identified a homozygous 2-bp deletion, c.2156_2157delAG, p(Lys645AlafsTer12), in a Bedouin family with DCM and hypertrabeculation, demonstrating that this mutation disrupted lysosomal function, leading to cardiomyopathy. Another study by Akins et al^2^ described a 21-year-old woman with DCM carrying 2 heterozygous variants in PLEKHM2, including a frameshift mutation and a splice variant, further supporting the gene’s role in cardiomyopathy. Our article further supports this association and highlights the need for continued research into the mechanisms by which PLEKHM2 mutation contributes to the advancement of DCM.

## Funding Support and Author Disclosures

The authors have reported that they have no relationships relevant to the contents of this paper to disclose.
